# Static, refractive and monolithic Fourier transform spectrometer: development and prototyping

**DOI:** 10.1038/s41598-023-51008-0

**Published:** 2024-01-12

**Authors:** Fabio Frassetto, Lorenzo Cocola, Paola Zuppella, Vania Da Deppo, Luca Poletto

**Affiliations:** Institute for Photonics and Nanotechnologies, National Research Council, Via Trasea 7, 35131 Padua, Italy

**Keywords:** Astronomical instrumentation, Astronomical instrumentation, Astronomical instrumentation

## Abstract

Static Fourier transform spectrometers are devices that can be realized as monolithic and compact assemblies. In the “grating-based” monolithic version, they are usually realized gluing together a beam-splitter with two reflective diffraction gratings using spacers as connecting elements. In this work we present the development and test of an alternative form of this kind of instrument in which the dispersive elements are Littrow’s prisms and are glued to the splitting element, forming in this way a robust and filled structure with no air gaps. The device can work in the visible/near infrared spectral region with a resolution power that varies across the spectral range due to the dispersion of the used glasses. The absence of hollow regions inside the monolithic block makes the device extremely robust and protects the optical surfaces inside the interferometer from possible contaminations. The device can be easily miniaturized, as it does not require spacers or structural elements other than just the optical parts. The tested instrument works in the 470–850 nm wavelength range with a variable resolution between 3000 and 300.

## Introduction

Depending on the working principle, spectrometers can be arranged in different types and subtypes. Recently, their possible miniaturization capability has entered as a merit parameter in the choice process^1,2^. Among all the techniques, in the last decades the spectroscopic methodology sometimes referred to as Spatial Heterodyne Spectroscopy (SHS), or Static Fourier Transform Spectroscopy (S-FTS)^3–8^, has gathered increasing visibility and interest. The methodology is nowadays well documented^9^ and we redirect the interested reader to the suggested references for a proper description of the technique. Here is an overview, sufficient to summarize the working principle of this technique: indicating with λ the wavelength of a generic monochromatic component of the analysed light, an interferometer produces a periodic pattern, called interferogram, in which the spatial frequency depends on λ. The central aspect of the SHS technique is the presence in the optical path of dispersive elements that produce a great change in the spatial frequency of the interferogram when λ differs from a particular λ_L_ called Littrow wavelength. This variation depends on the difference $\lambda -{\lambda }_{L}$, and for $\lambda ={\lambda }_{L}$ no interference fringes are produced. This effect explains the use of the term “heterodyne”.

This technique has been used in a wide range of applications^10–15^.

Traditionally, the most common dispersive elements are reflective diffractive gratings but, in general, every generic retro-dispersive element can be used. In a previous work we have analysed the use of Littrow prisms just to increase the signal to noise ratio^16^. The use of prisms paves the way to a second interesting possibility that is analysed in this work: gluing the prisms to the beam splitter without any spacer or supporting structure, thus realizing in this way a compact and extremely robust assembly without any air gap in the interferometric assembly. Here we report on the optical concept, the realization procedure and laboratory tests made on a demonstrative prototype. The absence of air gaps in the interferometric assembly, hereafter IA, prevents possible contaminations of the internal surfaces of the IA itself and makes it more robust with respect to framed configurations. Moreover, due to the frame-free assembly this device can be easily miniaturized.

The spectral dispersion produced by prisms cannot be as high as the one typical of gratings, nevertheless, the use of compound prisms^17–19^ can be a possible way to increase the spectral resolution of this kind of instruments.

To our knowledge, this optical configuration is new and never tested; we consider it of possible great interest in applications where low to medium resolution is required over an extended spectral interval together with the necessity to have a good light throughput. Some examples of similar applications, for astronomical or Raman spectroscopy, are reported in^20–23^. The mechanical solidity of the IA, the scalability and the simplicity of the optical layout make this optical concept interesting in a wide range of experimental cases. Possible applications benefiting from this technique include the ones related to the space environment, in particular the spectroscopy of planetary atmospheres or the realization of portable instrumentation, but also the realization of instruments for harsh environments.

## Optical design

**Figure 1:**
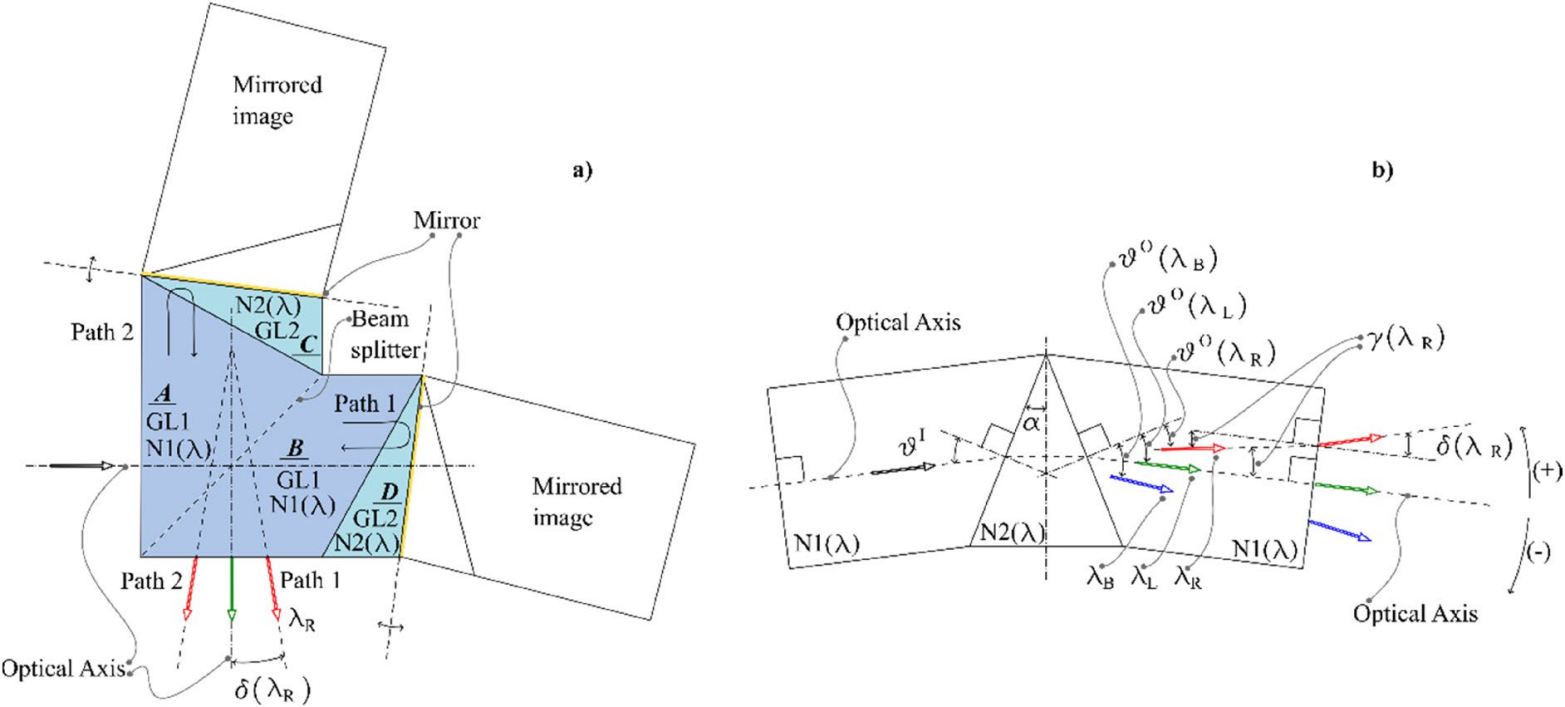
(**a**) Geometrical model of the interferometric assembly with the indications of its main characteristics. The green arrow represents the exiting direction for the Littrow wavelength, that is parallel to the optical axis. The arrows indicate the emerging directions of a generic wavelength ${\lambda \ne \lambda }_{L}$. (**b**) Equivalent fully transmissive model of each of the two paths of the interferometric assembly.

This section illustrates the analytical model used to evaluate the angular dispersion of a generic monochromatic component exiting the interferometric assembly. In Fig. 1a the coloured region representing the IA is composed of four physical elements named A, B, C and D. A and B constitute the beam splitter and are realized of an appropriate glass GL1. C and D are identical, and realized with a different glass, GL2. The radiation enters the beam-splitter orthogonally to the entering surface and no angular dispersion is introduced at this surface. The beam is separated at the beam splitter surface by a 50–50 non polarizing coating: a half is transmitted into path 1 and the other half reflected into path 2. On the two paths, two Littrow prisms, C and D, are glued to the beam splitter. The mirror coated surface on the two Littrow prisms introduces a mirror symmetry that permits to analyse each path as the equivalent fully transmissive optical system, as the one schematised in Fig. 1b. The choice of GL2 is an important degree of freedom that, joined with the choice of the apex angle of the two prisms and the inclination of the exit surfaces of the beam splitter, permits to tune the optical characteristic of the spectrometer.

**Figure 2:**
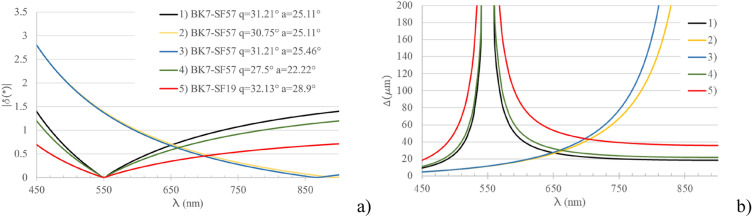
Effect of the variation of ${\vartheta }^{I}$, $\alpha$ and ${n}_{\mathrm{1,2}}\left(\lambda \right)$ on the working spectral range and dispersion. (1) is the configuration realized and analysed in this work. In (2) and (3) one of the two parameters ${\vartheta }^{I}$, $\alpha$ is varied. In (4) ${\vartheta }^{I}$, $\alpha$ are varied with the purpose of keeping ${\lambda }_{L}$ constant. (5) shows the effect of changing the glass GL2, SF57, with SF19 while ${\vartheta }^{I}$, $\alpha$ are tuned in order to maintain unaltered ${\lambda }_{L}$. (**a**) $\delta \left(\lambda \right)$ in the five considered cases in the 450–550 nm spectral region. (**b**) $\Delta \left(\lambda \right)$ evaluated at the FLP for the five different considered cases in the 450–850 nm spectral region.

Indicating with ${\vartheta }^{I}$ the incidence angle of the radiation at the interface between the elements A–C or equivalently B–D, $\alpha$ the apex angle of the Littrow prisms C and D and with ${n}_{\mathrm{1,2}}\left(\lambda \right)$ the refractive index of the two glasses used for the elements A and B and C and D respectively, the refracted angle ${\vartheta }^{O}\left(\lambda \right)$ can be evaluated as:

1 $${\vartheta }^{O}\left(\lambda \right)={{\text{sin}}}^{-1}\left(\frac{{n}_{2}\left(\lambda \right)}{{n}_{1}\left(\lambda \right)}{\text{sin}}\left(2\alpha -{{\text{sin}}}^{-1}\left(\frac{{n}_{1}\left(\lambda \right)}{{n}_{2}\left(\lambda \right)}{\text{sin}}\left({\vartheta }^{I}\right)\right)\right)\right).$$

As previously mentioned, there is a particular wavelength, called Littrow wavelength and indicated as ${\lambda }_{L}$, for which ${\vartheta }^{I}={\vartheta }^{O}\left(\lambda \right)$. For ${\lambda \ne \lambda }_{L}$ the angle that the radiation, still propagating in the elements A or B, forms with the optical axis is $\gamma \left(\lambda \right)={\vartheta }^{I}-{\vartheta }^{O}\left(\lambda \right)$. Exiting the IA, the radiation undergoes another refraction and the propagation angle, measured with respect the optical axis, changes from $\gamma \left(\lambda \right)$ to $\delta \left(\lambda \right)$ following the Snell’s law, Eq. (2).

2 $$\delta \left(\lambda \right)={{\text{sin}}}^{-1}\left({n}_{1}\left(\lambda \right){\text{sin}}\left(\gamma \left(\lambda \right)\right)\right)$$

For a generic wavelength ${{\lambda }_{R}>\lambda }_{L}$ $\delta \left({\lambda }_{R}\right)$ is considered positive, and the opposite for a generic wavelength ${{\lambda }_{B}<\lambda }_{L}$.

Considering a generic wavelength exiting the IA with an angle $\delta \left(\lambda \right)$, the two beams propagating along Path 1 and Path 2 produce at the fringe localization plane, hereafter FLP, spatial fringes with separation between the maxima given by the Eq. (3).

3 $$\Delta \left(\lambda \right)=\frac{\lambda }{2{\text{sin}}\left(\delta \left(\lambda \right)\right)}$$

The FLP can be visualized backpropagating two rays exiting the IA and produced by the same incoming ray and looking at the plane where they recombine.

Using as freedom degrees the values of ${\vartheta }^{I}$, $\alpha$ and ${n}_{\mathrm{1,2}}\left(\lambda \right)$, spectrometers working in different spectral regions and with different resolutions can be designed. As an example, in Fig. 2 the values of $\delta \left(\lambda \right)$ and $\Delta \left(\lambda \right)$ are evaluated using Eqs. (1–3) for five different cases: cases 1 to 4 show the effect of the change in the values of ${\vartheta }^{I}$ and $\alpha$; case 5 gives an indication on how the change in the glass used for the prisms C and D changes the dispersion capability of the IA.

### The prototype instrument

With the purpose of realizing and testing a demonstrative instrument we choose to work close to the centre of the visible band by imposing ${\lambda }_{L}$= 550 nm; this permits to easily explore the behaviour of the spectrometer both at higher and lower wavelengths with respect to ${\lambda }_{L}$. Moreover, we planned to test the instrument over the 625–792 nm band (a region where we could evaluate several atmospheric absorption features as references). In order to keep the longest wavelength in the spectrum far from the Nyquist limit (where instrument performance may be affected by aberrations of the imaging system) we have chosen ${\vartheta }^{I}$ = 31.21° and $\alpha$ = 25.11°.

**Figure 3:**
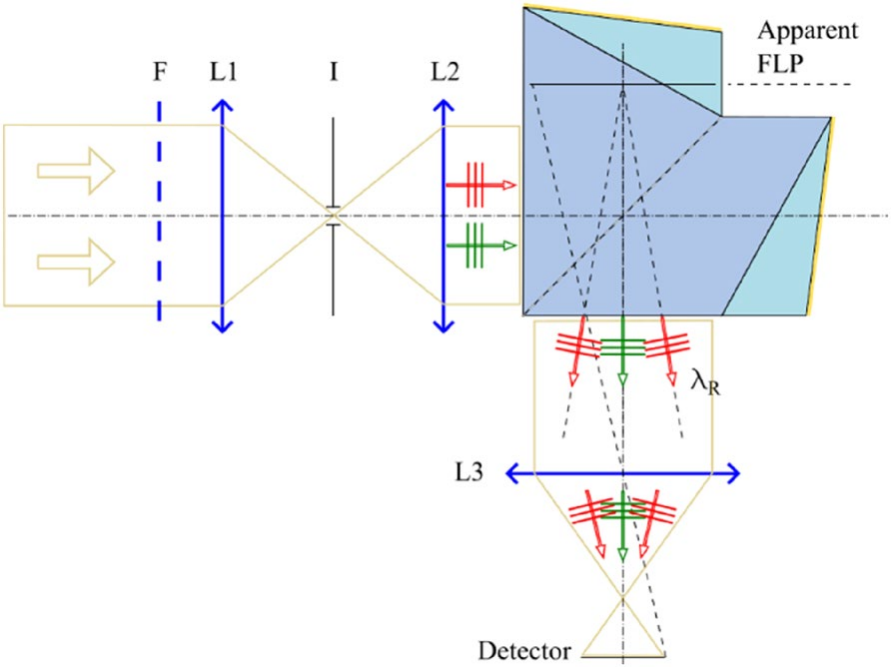
Layout of the realized instrument. F, band pass filter; L1, condensing lens; I, circular aperture; L2, collimator; L3, imaging system. Apparent FLP indicated location is the position as seen by the imaging system in an average focusing condition.

The instrument layout is shown in Fig. 3. The light entering the instrument is spectrally limited by the band pass filter, F. L1 is a condenser lens and focalizes the radiation in a circular aperture, I, used to limit the angular acceptance of the spectrometer. L2 is a commercial optical system used to collimate the light before the IA. At the exit port of the IA a second commercial optical system, L3, conjugates the FLP with the detector plane.

**Figure 4:**
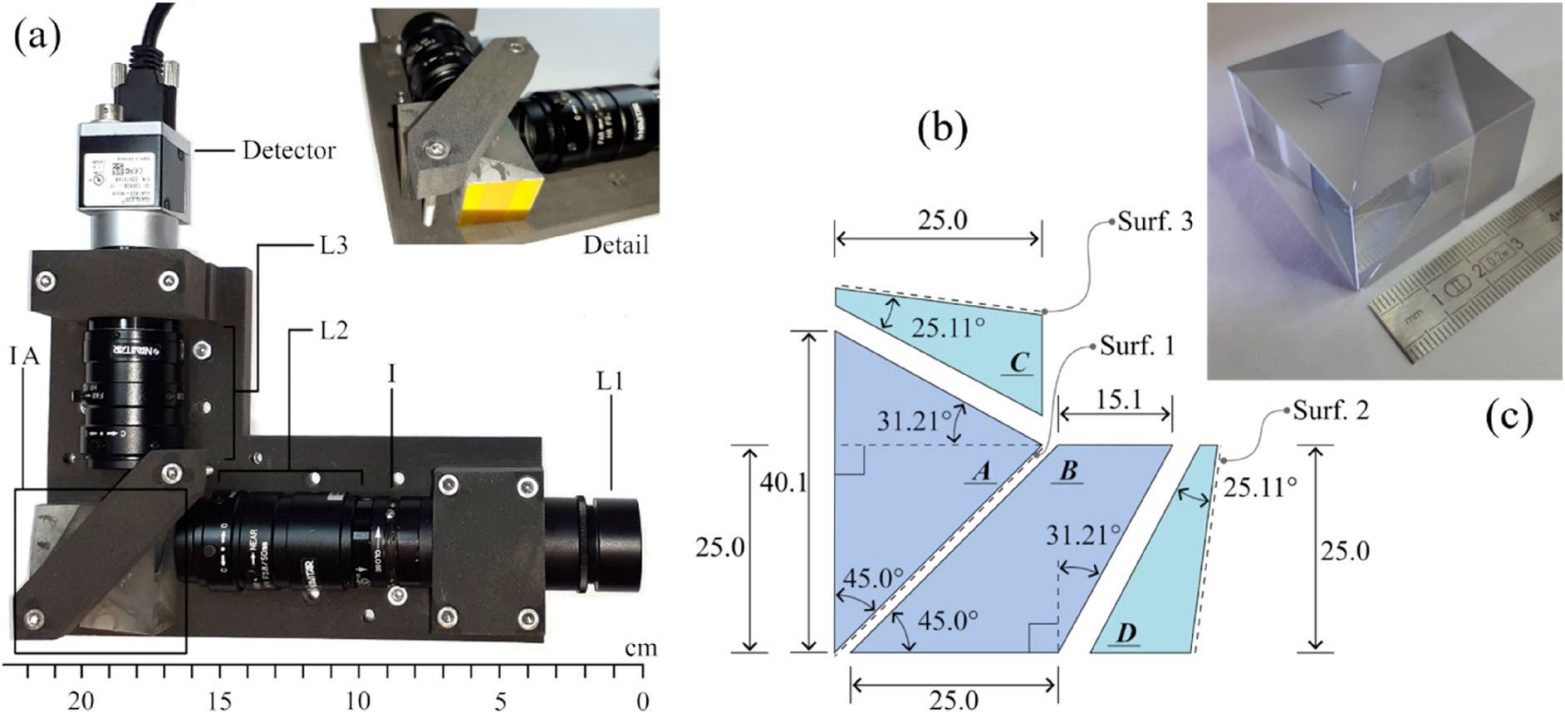
The instrument as realized for the tests. (**a**) Image of the spectrometer. All the optical elements are installed on the 3D printed frame. The name convention is the same as in Fig. 3. The insight shows a different view of the IA and its clamping system. (**b**) Geometrical dimensions of the IA. (**c**) Detail of the interferometric assembly.

The physical realization of the instrument is presented in Fig. 4. Figure 4a shows the whole instrument and a detail of the IA clamping system. Figure 4b illustrates the geometrical dimensions of IA. Surface 1 is the splitting surface and realized on the element A. Surfaces 2 and 3 are aluminum coated. All the optical elements are installed on a 3D printed structure made of sintered PA12. Figure 4c is a picture of the bare IA with a ruler as scale element. The main parameters of the instrument are listed in Table 1. The mass of the IA is 87 g and the envelope of a rectangular cuboid containing IA is 43 mm × 43 mm × 25 mm.

**Table 1:** List of the main characteristics of the spectrometer.

L1	Diameter 25 mm, focal length 75 mm
L2	Navitar F2.8/50 mm
L3	Navitar F2/35 mm
I	Variable diameter 0.5–3 mm
Detector	Basler aca 1920–40 um, 1920 px × 1200 px, 5.86 µm × 5.86 µm, mono, CMOS

### Assembling of the interferometer

**Figure 5:**
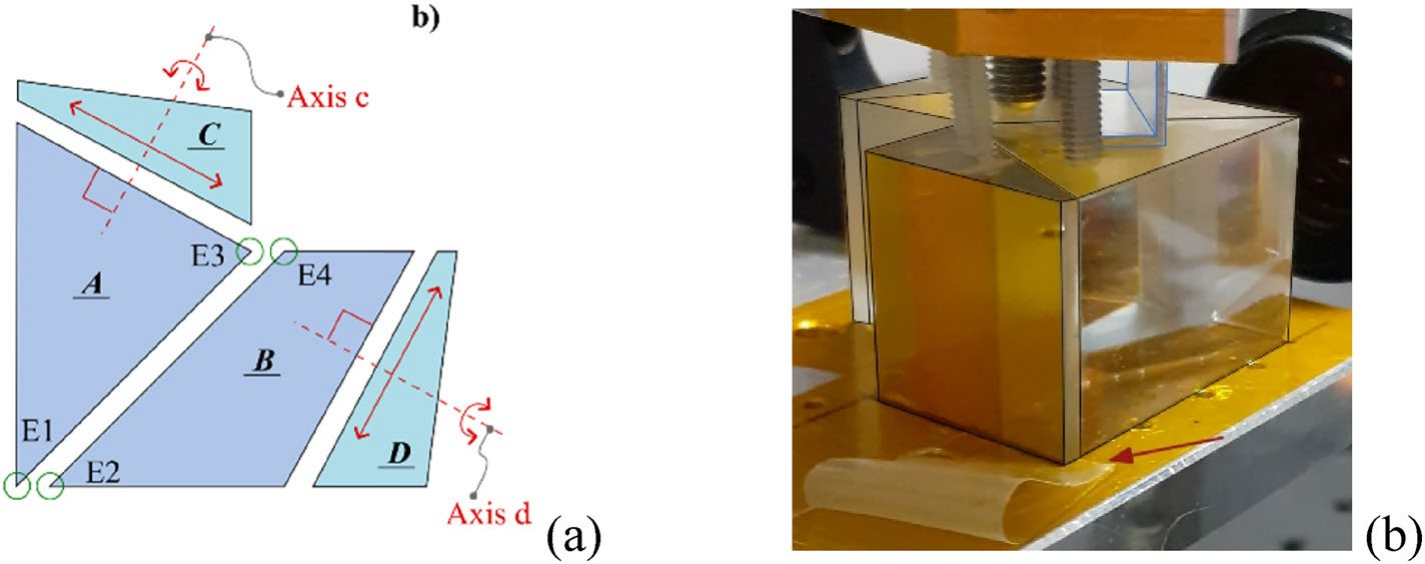
IA alignment tests. (**a**) Alignment points (green circles) and movements used as freedom degrees for the remaining parts C and D. (**b**) The IA on the alignment mount: the black lines are used to highlight the glass structure. In blue the metallic structure used to link two parts, in this particular case the parts A and B. The red arrow indicates the shim. The screws on top are used to maintain in place the optical group.

The gluing procedure of the IA has been carefully evaluated. Several tests intended to verify the most effective gluing sequence have been performed using an index matching liquid (Cargille Immersion Liquid, Code 81520 BK-7 Matching Liquid, Product code: 19586) instead of the glue. Each sequence has been done selectively locking two elements and using the remaining two as compensators for the equalization of the optical path length along the two optical paths and to provide degrees of freedom to align the interference pattern orthogonally to the dispersion plane. Figure 5a shows the scheme of the IA with the indication of the alignment points and the movements used in the alignment procedure, Fig. 5b the IA on the alignment platform.

**Figure 6:**
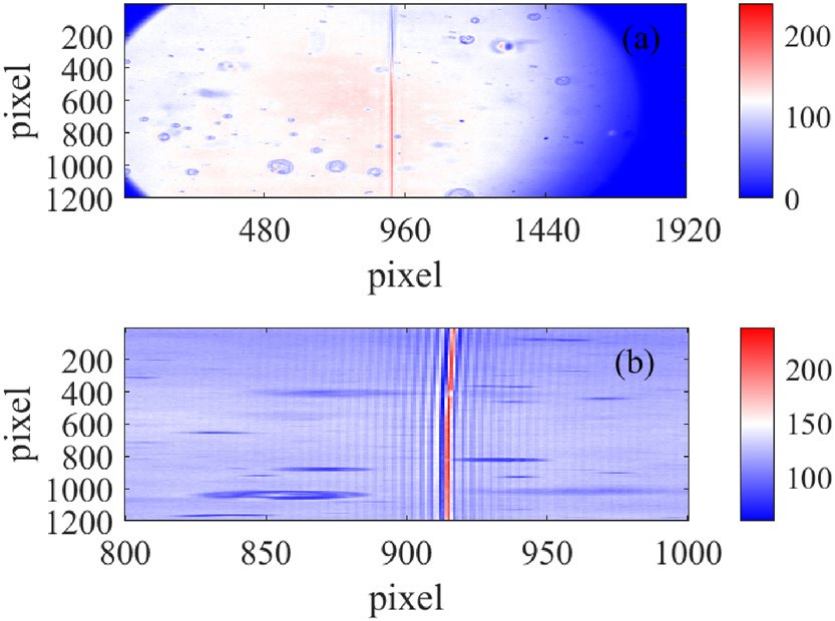
Interferogram corresponding to an almost optimal alignment. (**a**) The full interferogram, the bubbles are due to air inclusions in the oil. (**b**) Zoom in the region 800–1000 pixels.

An almost optimal alignment is reachable, as illustrated in Fig. 6 where an example of a nearly optimally aligned interferogram, acquired during these tests with the matching liquid, is presented.

The adopted procedure is finally described here. The two BK7 elements, A and B in Fig. 5a, are initially glued aligning the edges E1 with E2 and E3 with E4. This alignment is purely visual, no optical references are monitored in this phase. Subsequently, the two prisms C and D are joined to the assembly. These two optical elements can be translated along the directions indicated with a red arrow in Fig. 5a. These translations are used to equalize the optical path along Path 1 and Path 2. Having two translations to act on, this equalization can be tuned to centre the interferogram of a broad band test source in the middle of the field of view. The edge originated in E3–E4, when part A and B are glued together, only permits movements in one direction. Rotations around the axes c and d in Fig. 4a are realized using shims, see Fig. 4b, and are used to align the interference fringes pattern orthogonally to the dispersion plane.

The index matching liquid used for tests was then carefully removed with acetone before the final gluing, which was then done in a permanent way.

The used glue is the Optical Adhesive 61, NOA61, from Norland Products Inc., and is cured by using a UV diode lamp (365 nm) after a final interferometric check of the alignment.

The final alignment is not optimal as the one shown in Fig. 6 but is acceptable and has produced the results presented in the next paragraph.

### Spectral calibration

**Figure 7:**
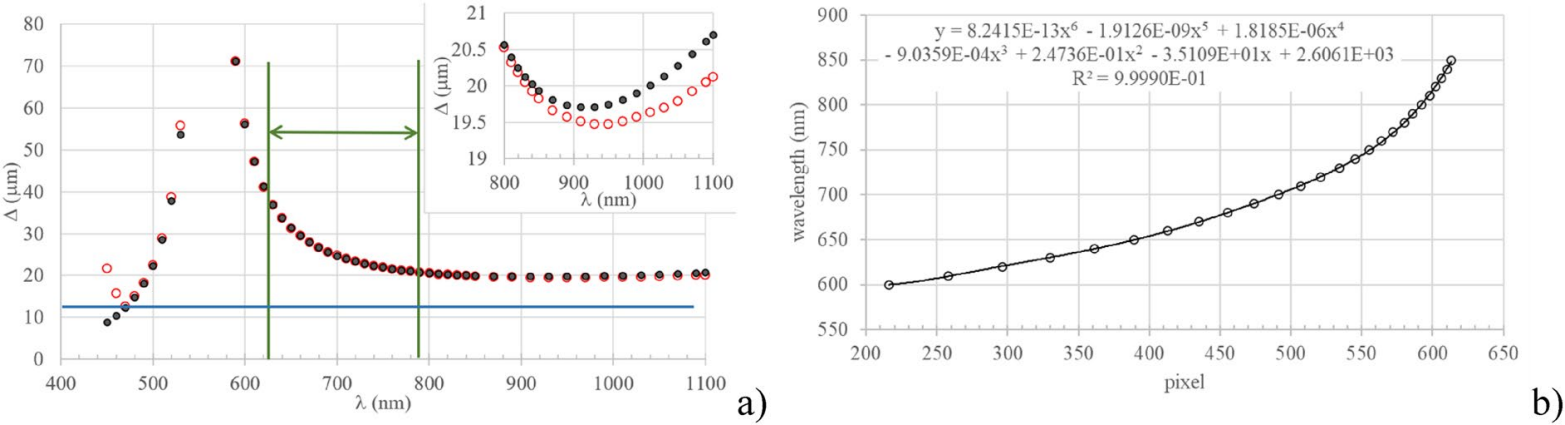
Spectral calibration of the spectrometer. (**a**) Fringe separation at the FLP for a monochromatic component. Empty red circles are from measured data, black dots represent values obtained from the analytical model. The discrepancy between measured and simulated values that appears at wavelengths smaller than 470 nm is due to aliasing. The horizontal blue line, positioned at 12 µm, indicates the two pixels limit. The secondary plot shows a zoom into the region 800–1100 nm, where a minimum for the curve appears at about 930 nm. The spectral interval delimited with two vertical green lines is the working spectral region used in the final part of the paper. (**b**) Calibration plot: in abscissa the pixel coordinate in the Fourier domain, in ordinate the corresponding wavelength. The equation on top is a polynomial fit having the indicated coefficient of determination.

The instrument has been calibrated using a monochromator as an illumination system. While varying the wavelength across (and beyond) the working spectral region, various interferograms have been acquired and the corresponding spectra have been reconstructed. Figure 7a illustrates the variation of the fringe separation at the FLP (calculated considering the magnification factor of L3) for a generic monochromatic component. The black dots and the empty red circles represent the values calculated from the analytical model and those calculated from experimental acquisitions respectively. The horizontal blue line, set at 12 µm, is used to identify the Nyquist limit corresponding to two sample intervals. The vertical green lines show the spectral region investigated later in the article. It is interesting to observe that at 930 nm there is a minimum in the variation law of Δ: for wavelength larger than 930 nm the term dominating in the fraction of Eq. 3 is the numerator, and the curve becomes increasing. Figure 7b presents the calibration fit of the instrument in the range 600–850 nm.

## Experimental verification

**Figure 8:**
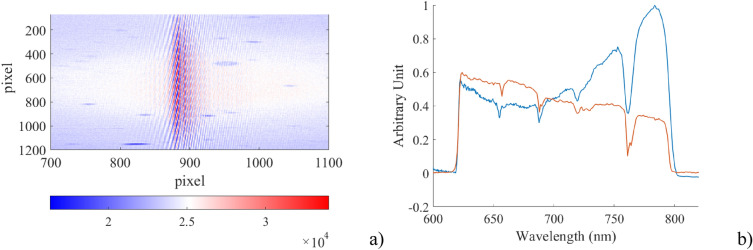
Spectrum of the diffuse skylight. (**a**) The acquired interferogram. For clarity of representation, the image is 1200 pixels high and 400 pixels wide. (**b**) Comparison between measurements: the red curve is the spectrum acquired with the reference spectrometer, the blue one is the spectrum originated with the spectrometer under test and originated from the interferogram on the (**a**) panel. All the spectra have been acquired using an interferential filter in front of the spectrometers limiting the wavelength range to the 625–792 nm interval.

In order to test the instrument with a diffuse, extended and broad band source, we have acquired the diffuse skylight during a mostly sunny day and compared the results with a spectrum obtained soon after using a commercial spectrometer (Admesy Hera), while pointing at the same sky region. This comparison is only qualitative due to the source variability originated both from the clouds movements and from the pointing error between the two instruments. Moreover, we expect a change in the instrumental response associated with a variation in the FLP distance (as a function of wavelength) that L3 focuses on the detector plane. As we focus the imaging optics on a particular FLP distance, we obtain a spectral response peaking at that corresponding wavelength. The circular aperture was set to 3 mm as this setting showed no noticeable degradation of the spectral resolution. These phenomena are not discussed in this work and need further investigations. To avoid the aliasing effect, we have installed an interferential bandpass filter in front of both the spectrometers. This filter (Semrock 709 167) limits the spectral band of the analysed radiation to the interval 625–792 nm: this is the spectral region illustrated in Fig. 7a with the two vertical green lines. Compared to the best obtained result of Fig. 6, in Fig. 8 the fringes of the interferogram are slightly tilted. This effect does not cause an appreciable degradation in the performance of the instrument.

## Conclusions and discussion

In this paper, a monolithic and static Fourier transform spectrometer has been realized and tested. The instrument uses two Littrow’s prisms as retro-dispersive elements, no diffraction gratings are used. The interferometric part is realized gluing the two prisms to the beam splitter. As a result, the interferometer has no hollow volumes inside and all the surfaces between the splitting and recombination of the wavefront in the interferometer are protected. The dispersion of glasses makes the resolution extremely variable across the spectral working range (from 300 to 3000); we show how the degrees of freedom in the optical design can be used to tune the instrument operation and performance. Thanks to the robustness of the IA the realization of the instrument on a fully 3D printed plastic structure has been possible. The absence of hollow parts gives to the IA assembly the same rigidity of a typical cube beam-splitter, making it suitable for harsh environment: as an example those in which vibrations or a high level of dust contamination can be an issue. Moreover, gratings can act as a source of diffuse light, unless expensive gratings are used, while the surfaces of the Littrow’s prisms can be more easily realized with a low roughness.

We consider this optical implementation of interest for specific applications both in the research and industrial fields, especially when medium resolution is required on the VIS–NIR spectral region. Moreover, we think that this work can drive further investigations on open questions such as the field widening techniques and the focusing depth of the fringe localization plane.

## Data Availability

The datasets used and analysed during the current study are available from the corresponding author upon reasonable request.

## References

[1] Yang Z, Albrow-Owen T, Cai W, Hasan T (2021). Miniaturization of optical spectrometers. Science.

[2] Barnett PD, Angel SM (2017). Miniature spatial heterodyne raman spectrometer with a cell phone camera detector. Appl. Spectrosc..

[3] Harlander, J. M. Spatial heterodyne spectroscopy: Interferometric performance at any wavelength without scanning. Thesis (Ph.D.) University of Wisconsin, Madison, https://ui.adsabs.harvard.edu/abs/1991PhDT........62H (1991).

[4] Harlander J, Roesler F, Cardon J, Englert C, Conway R (2002). Shimmer: A spatial heterodyne spectrometer for remote sensing of Earth’ middle atmosphere. Appl. Opt..

[5] Englert CR, Harlander JM, Brown CM, Marr KD (2015). Spatial heterodyne spectroscopy at the Naval Research Laboratory. Appl. Opt..

[6] Englert, C. R., Harlander, J. M., Harding, B. J. & Marr, K. D. Low-signal phase shift: Characterizing an unexpected detector deterioration of the ICON/MIGHTI instrument. In *Optica Sensing Congress 2023 (AIS, FTS, HISE, Sensors, ES), Technical Digest Series* (Optica Publishing Group, 2023), paper FTu5B.4. 10.1364/FTS.2023.FTu5B.4.

[7] Kaufmann M (2018). A highly miniaturized satellite payload based on a spatial heterodyne spectrometer for atmospheric temperature measurements in the mesosphere and lower thermosphere. Atmos. Meas. Tech..

[8] Lenzner M, Diels J-C (2016). Concerning the spatial heterodyne spectrometer. Opt. Express.

[9] Zhang W-L (2021). Research status of spatial Heterodyne spectroscopy - A review. Microchem. J..

[10] Yi Y, Zhang S, Liu F, Zhang Y, Yi F (2017). Laboratory fabrication of monolithic interferometers for one and two-dimensional spatial heterodyne spectrometers. Opt. Express.

[11] Wu X, Tan Y, Yi Y, Zhang Y, Yi F (2019). Two-dimensional spatial heterodyne spectrometer for atmospheric nitrogen dioxide observations. Opt. Express.

[12] Burke MG, Fonck RJ, Mckee GR, Winz GR (2023). Spatial heterodyne spectroscopy for fast local magnetic field measurements of magnetized fusion plasmas. Rev. Sci. Instrum..

[13] Zhang WL (2022). Experimental realization of visible gas sensing technology based on spatial heterodyne spectroscopy. Sci. Rep..

[14] Foster MJ, Storey J, Zentile MA (2017). Spatial-heterodyne spectrometer for transmission-Raman observations. Opt. Express.

[15] Kaufmann M (2019). On the assembly and calibration of a spatial heterodyne interferometer for limb sounding of the middle atmosphere. CEAS Space J..

[16] Frassetto F, Cocola L, Zuppella P, Da Deppo V, Poletto L (2021). High sensitivity static Fourier transform spectrometer. Opt. Express.

[17] Hagen N, Tkaczyk T (2011). Compound prism design principles, I. Appl. Opt..

[18] Hagen N, Tkaczyk T (2011). Compound prism design principles, II: Triplet and Janssen prisms. Appl. Opt..

[19] Hagen N, Tkaczyk T (2011). Compound prism design principles, III: Linear-in-wavenumber and optical coherence tomography prisms. Appl. Opt..

[20] Ammler-von Eiff M, Sebastian D, Guenther EW, Stecklum B, Cabrera J (2015). The power of low-resolution spectroscopy: On the spectral classification of planet candidates in the ground-based CoRoT follow-up. Astron. Nachr..

[21] Boonsit S, Kalasuwan P, van Dommelen P, Daengngam C (2021). Rapid material identification via low-resolution Raman spectroscopy and deep convolutional neural network. J. Phys: Conf. Ser..

[22] Oke JB (1995). The keck low-resolution imaging spectrometer. PASP.

[23] Zemcov M (2013). The cosmic infrared background experiment (CIBER): A sounding rocket payload to study the near infrared extragalactic background light. ApJS.

